# Low-Velocity Penetrating Cranial Trauma With a Retained Axe: Surgical Management and Operative Challenges in a Civilian Assault Case

**DOI:** 10.7759/cureus.105822

**Published:** 2026-03-25

**Authors:** Jason Golmei, Shubham Kaushal, Hanuman Prasad, Bal Krishna Ojha, Shweta Dubey

**Affiliations:** 1 Neurosurgery, King George's Medical University, Lucknow, IND; 2 Radiodiagnosis, King George's Medical University, Lucknow, IND

**Keywords:** dural repair, low-velocity cranial trauma, nonmissile penetrating head injury, penetrating brain injury, retained intracranial foreign body

## Abstract

Penetrating brain injury (PBI) is a rare but potentially life-threatening form of traumatic brain injury characterized by violation of the skull and dura by a foreign object. Civilian penetrating head injuries caused by low-velocity objects such as knives, rods, or tools are uncommon and present unique diagnostic and surgical challenges. We report the case of a 23-year-old man who presented with a retained axe embedded in the right parietal region following an assault. On arrival, the patient was conscious with a Glasgow Coma Scale score of 15 but had left-sided hemiparesis. Non-contrast computed tomography (CT) of the head demonstrated a depressed right parietal fracture with a penetrating metallic foreign body extending intracranially. Emergency surgical exploration with controlled removal of the retained object, debridement of devitalized tissue, and dural repair was performed. The postoperative course was uneventful, and the patient was discharged on the tenth postoperative day with a healthy wound and stable neurological status, although residual hemiparesis persisted. At 15-day follow-up, no new neurological deficits were noted. This case highlights the importance of careful imaging evaluation, controlled extraction of penetrating objects along their trajectory, and meticulous dural repair. However, given the single-case nature and short follow-up, these findings should be interpreted with caution. In this patient, non-contrast CT was deemed sufficient as the trajectory was away from major intracranial vascular structures and no radiological indicators of vascular injury were identified.

## Introduction

Traumatic brain injury (TBI) remains a major global health problem, with millions of cases occurring annually worldwide [1]. Penetrating brain injury (PBI) represents a smaller but particularly severe subset of TBI in which a foreign object breaches the skull and dura mater, often causing direct parenchymal damage and vascular injury [2]. Civilian PBIs are most commonly caused by high-velocity projectiles such as firearms; however, low-velocity penetrating injuries caused by sharp or pointed objects are also encountered, especially in assault-related trauma [3].

Low-velocity PBIs differ from ballistic injuries in that the damage is typically confined to the tract of penetration without extensive cavitation, often resulting in better survival outcomes when managed appropriately [4]. Despite their relatively localized injury pattern, retained foreign bodies pose significant surgical risks, including hemorrhage, vascular injury, infection, and cerebrospinal fluid (CSF) leakage [5].

Imaging evaluation plays a critical role in surgical planning. Computed tomography (CT) is considered the imaging modality of choice for identifying the trajectory of the penetrating object, associated fractures, and intracranial hemorrhage [6]. In selected cases where vascular injury is suspected, CT angiography or digital subtraction angiography may be required to evaluate major intracranial vessels [7].

The primary goals of surgical management include removal of the foreign body under controlled conditions, debridement of devitalized tissue, hemostasis, and watertight dural closure to prevent infection and CSF leakage [8]. Early surgical intervention combined with broad-spectrum antibiotics and seizure prophylaxis is recommended to reduce morbidity [9].

We present a rare case of penetrating cranial trauma with a retained axe lodged in the parietal region, highlighting not only its rarity but also the unique operative challenges related to stabilization and controlled extraction of a heavy retained object, as well as intraoperative decision-making in the absence of vascular imaging.

## Case presentation

A 23-year-old man presented to the emergency department following a physical assault in which an axe was lodged into his skull. The injury occurred approximately 10 hours prior to admission. The patient was initially treated at a district hospital, where he received tetanus prophylaxis and initial wound care, and was subsequently referred to our tertiary care center for further management. On arrival, the patient was hemodynamically stable with no evidence of associated systemic injuries. There was no history of loss of consciousness or seizure activity reported prior to presentation.

**Figure 1:**
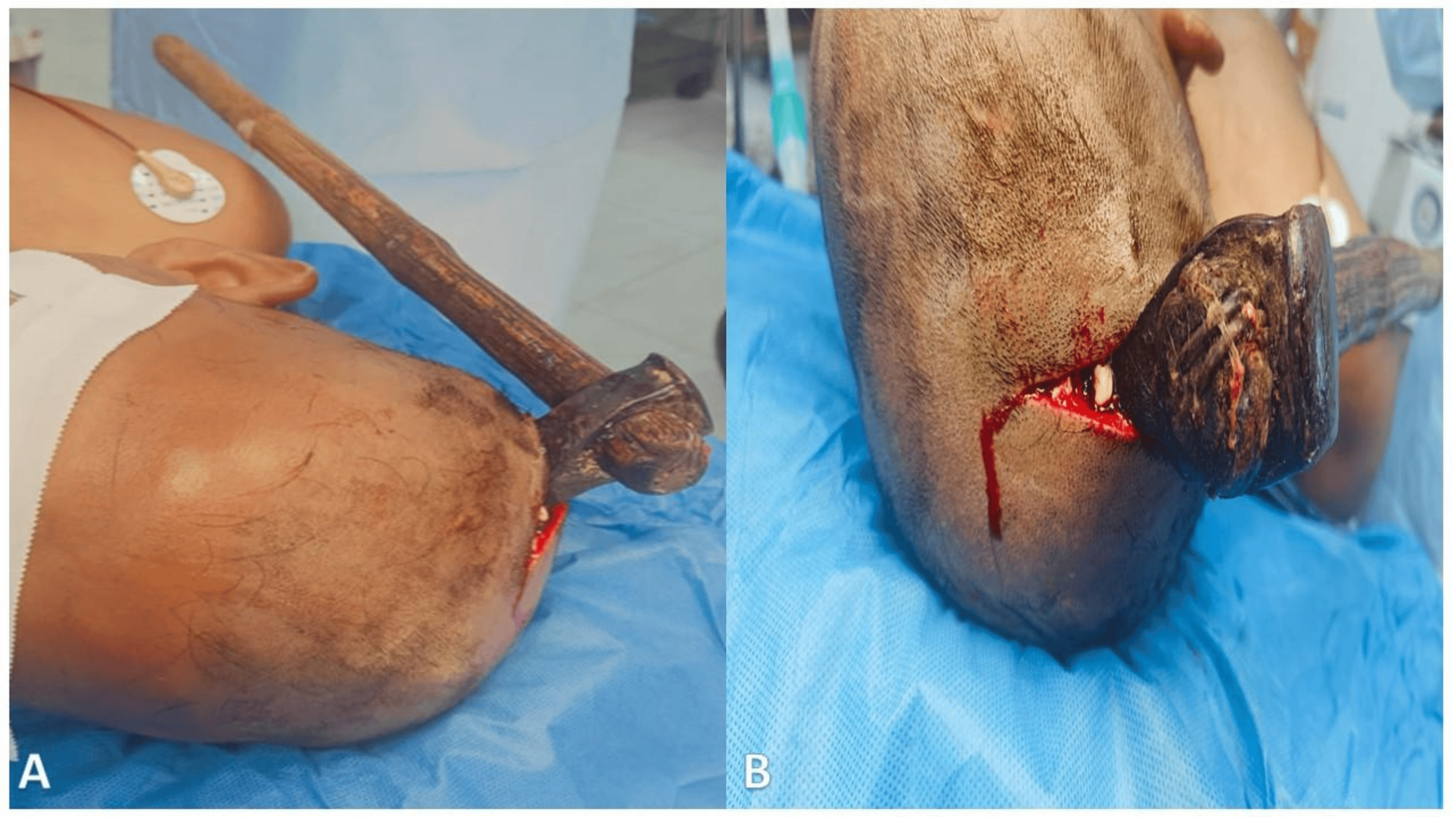
Clinical presentation showing the retained axe in the right parietal region. (A) Lateral view demonstrating the axe impaling the right parietal region of the skull following assault. (B) Close-up view of the entry site showing the axe head embedded in the scalp with surrounding laceration, extrusion of brain parenchyma, and active bleeding.

On examination, the patient had a Glasgow Coma Scale score of E4V5M6. Neurological examination revealed left-sided hemiparesis with muscle power of 1/5 (Medical Research Council grading) [10]. Local examination demonstrated the blade of an axe lodged in the right parietal region with surrounding scalp laceration and extrusion of brain parenchyma (Figure 1).

Imaging

**Figure 2:**
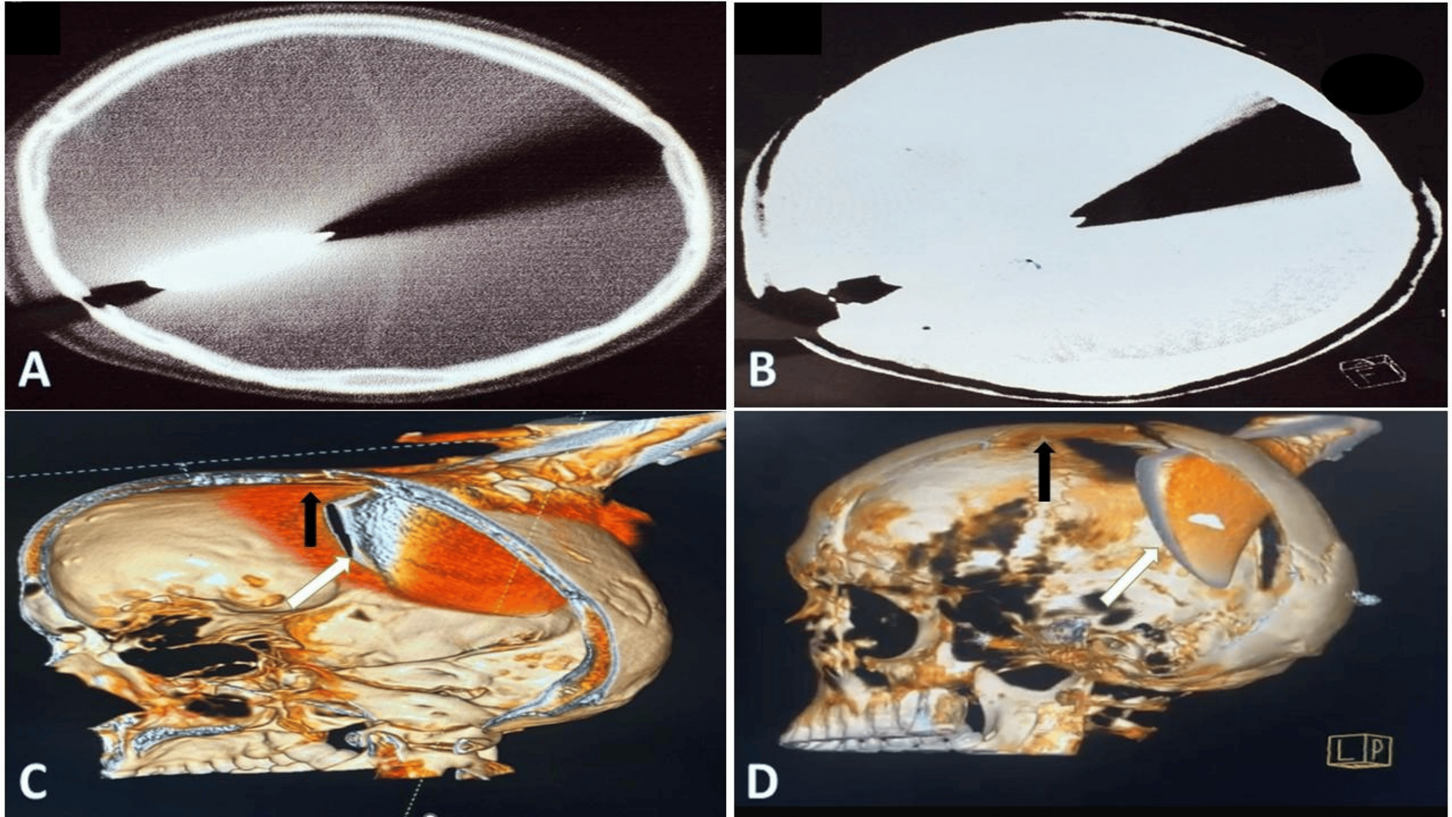
Preoperative computed tomography (CT) and three-dimensional reconstruction demonstrating the intracranial trajectory of the retained axe. (A) Axial CT scan (brain window) showing the metallic foreign body penetrating the right parietal bone with associated streak artifact. (B) Axial CT scan (bone window) demonstrating a depressed fracture of the right parietal bone with the foreign body extending intracranially. (C) Three-dimensional CT reconstruction (lateral view) demonstrating the trajectory of the axe penetrating the cranial vault. The black arrow indicates the midline, while the white arrow indicates the distal end of the axe blade extending inferior to the midline and away from the superior sagittal sinus. (D) Three-dimensional CT reconstruction highlighting the entry site at the right parietal bone and the orientation of the embedded axe head. The black arrow indicates the cranial midline. The white arrow denotes the distal end of the retained axe blade extending inferior to the midline, directed away from the superior sagittal sinus.

Non-contrast CT scan of the head demonstrated a depressed fracture of the right parietal bone with a retained metallic foreign body penetrating the cranial vault (Figure 2). The trajectory of the object extended intracranially from the parietal region. CT scan of the cervical spine revealed no associated cervical injury. CT angiography was considered; however, it was not performed as the trajectory of the foreign body was away from major intracranial vessels and the superior sagittal sinus, and there were no radiological indicators of vascular injury.

Surgical management

**Figure 3:**
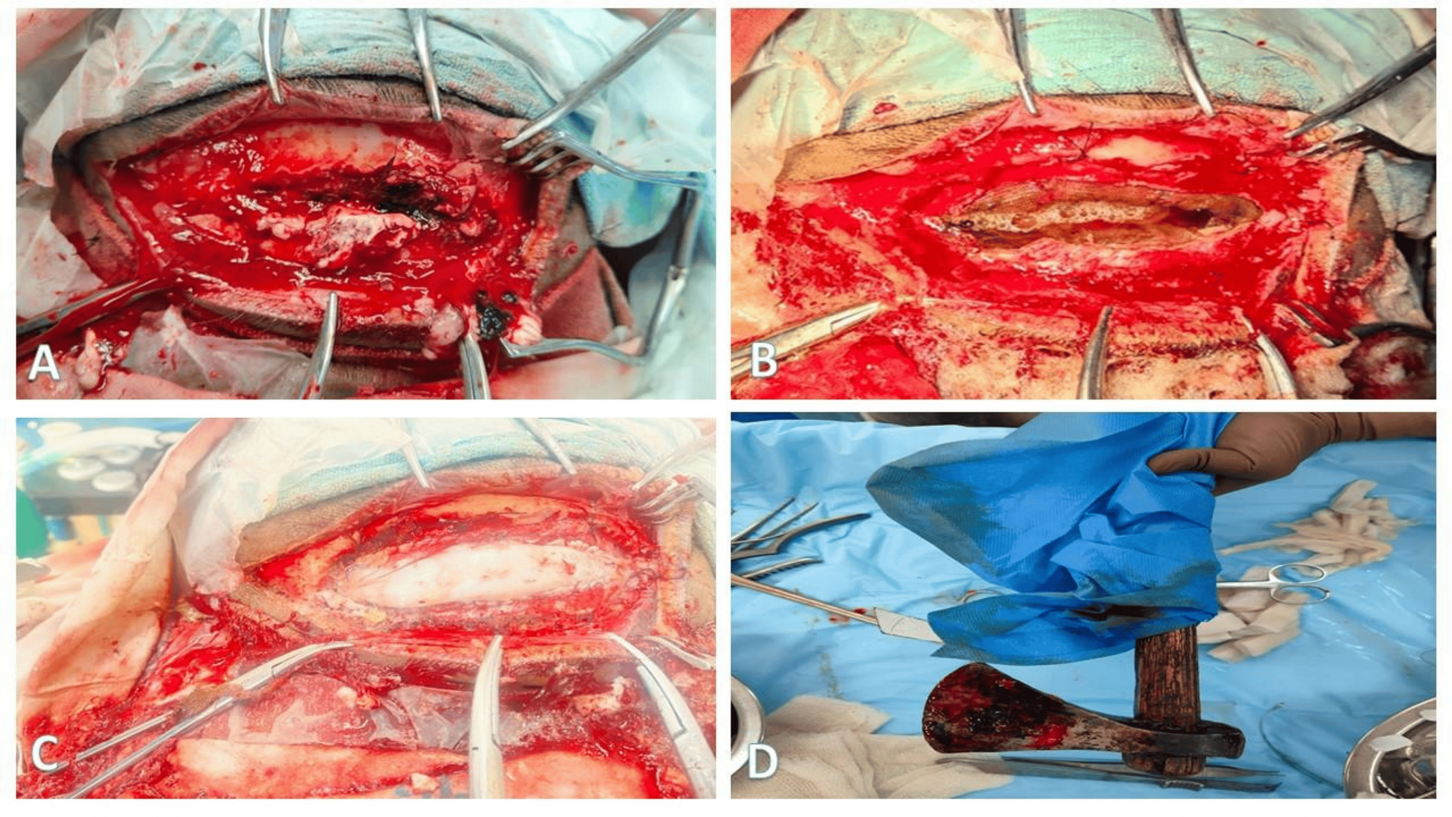
Intraoperative findings and surgical management. (A) Intraoperative view after removal of the axe demonstrating the entry site and surrounding fractured bone fragments. (B) Exposure of the cranial defect following debridement of contaminated tissue and irrigation with hydrogen peroxide and antibiotic-mixed saline. (C) Intraoperative view following repair of the dural rent using a tensor fascia lata (TFL) graft. (D) Extracted axe following controlled removal along the original trajectory.

The patient was taken for emergency surgery under general anesthesia. A linear incision was extended on either side of the wound to adequately expose the fracture margins. Devitalized bone fragments were removed, and the surrounding tissue was carefully debrided (Figure 3).

The foreign body was stabilized during the procedure to prevent inadvertent movement. Controlled removal of the axe was performed along the same trajectory to avoid secondary brain injury. After removal, a dural defect measuring approximately 6×3 cm was identified.

**Figure 4:**
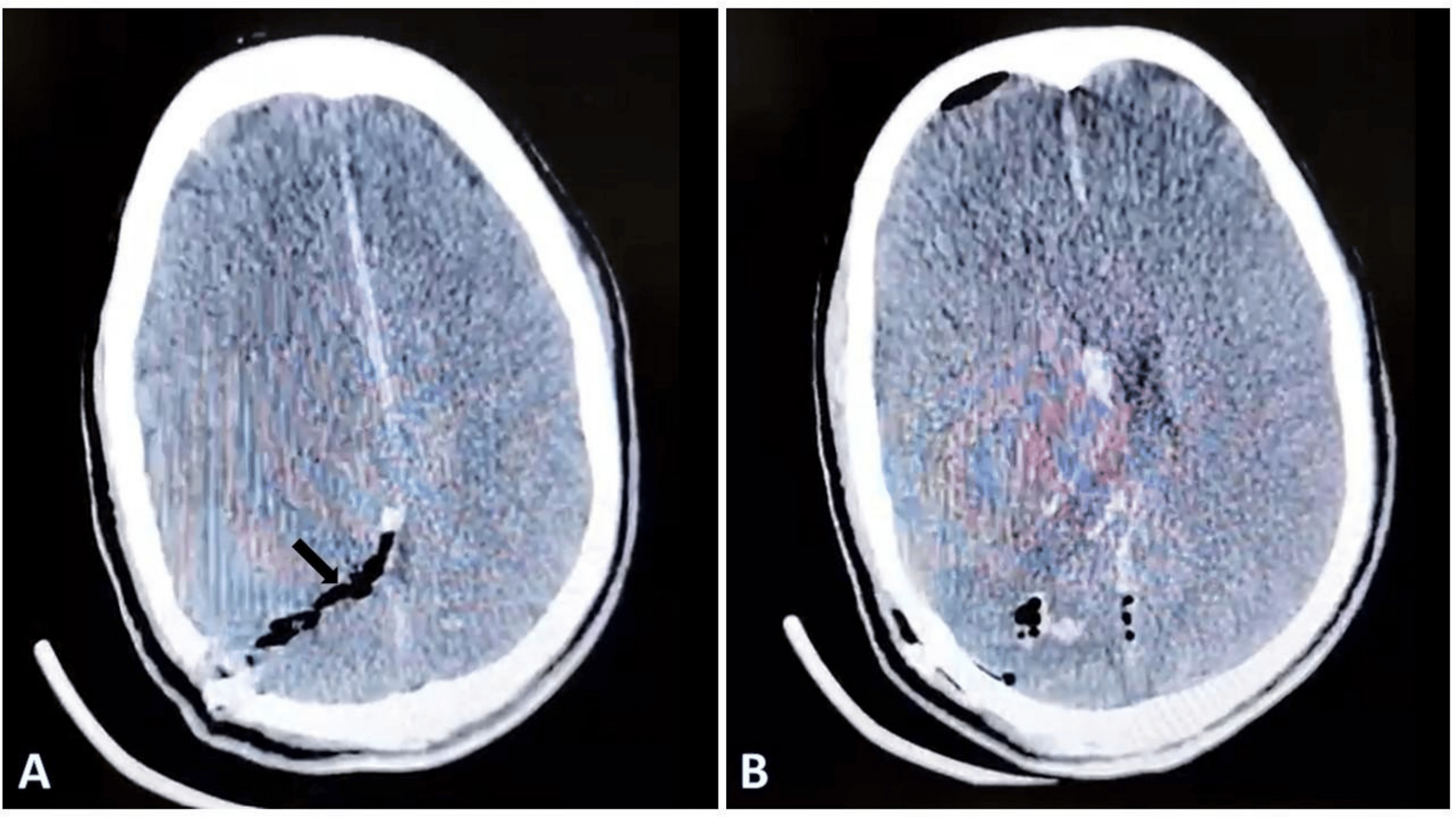
Postoperative computed tomography demonstrating intracranial changes after removal of the retained axe. (A) Axial postoperative CT scan demonstrating the postoperative tract with residual intracranial air and hemorrhagic contusion along the trajectory (arrow). (B) Axial postoperative CT scan at a lower section showing postoperative changes without significant mass effect.

Thorough irrigation with hydrogen peroxide and antibiotic-mixed saline was performed. The dural defect was repaired using a tensor fascia lata (TFL) graft with surgical adhesive to achieve watertight closure. Hemostasis was secured, and an intraoperative lumbar drain was placed. Broad-spectrum intravenous antibiotics and seizure prophylaxis were administered perioperatively. The lumbar drain was placed to reduce the risk of postoperative cerebrospinal fluid leakage and to facilitate dural healing. Postoperative Non-contrast CT scan of the head was satisfactory (Figure 4).

Postoperative course

The postoperative period was uneventful. The patient remained hemodynamically stable with no new neurological deficits. The previously noted left-sided hemiparesis persisted but did not worsen. The surgical wound healed well without evidence of infection or CSF leak. The patient was discharged on the tenth postoperative day with a healthy wound and stable neurological status. At 15-day follow-up, the patient remained clinically stable with persistent hemiparesis without significant improvement, and no new neurological deficits were noted.

## Discussion

PBIs account for approximately 10% of traumatic brain injuries in civilian populations and are associated with significant morbidity and mortality [11]. Low-velocity PBIs are typically caused by objects such as knives, metal rods, tools, or wooden sticks and usually result in more localized injury compared with high-velocity ballistic trauma [12]. Similar injuries caused by unusual penetrating objects, including knives, agricultural tools, and construction rods, have also been reported in civilian trauma literature [13]. Penetrating cranial trauma caused by large agricultural tools such as axes is exceedingly rare, and the presence of a retained heavy weapon embedded in the cranial vault presents unique operative challenges related to stabilization, controlled extraction, and prevention of secondary vascular injury. The extent of neurological damage in penetrating head injury depends on multiple factors, including the trajectory of the object, the involvement of vascular structures, and the initial neurological status of the patient [14]. The Glasgow Coma Scale score at presentation remains one of the most important predictors of outcome in patients with penetrating head trauma [15].

Radiological evaluation is essential prior to surgical intervention. CT scanning remains the primary imaging modality because it allows rapid assessment of the trajectory of the object, associated fractures, intracranial hemorrhage, and presence of pneumocephalus [6]. In cases where the penetrating tract is near major vessels or dural venous sinuses, CT angiography may help identify vascular injury or pseudoaneurysm formation [7].

Surgical removal of retained intracranial foreign bodies must be performed carefully to avoid catastrophic hemorrhage. Objects embedded in the cranial cavity may tamponade injured vessels, and premature removal outside the operating room may lead to uncontrolled bleeding [16]. A key principle in penetrating cranial trauma is that retained objects should never be removed outside the operating room, as they may tamponade injured vessels and prevent catastrophic hemorrhage. Therefore, extraction should be performed under direct visualization with adequate exposure of the surrounding bone and dura.

Debridement of contaminated tissue and removal of devitalized bone fragments are essential steps in preventing infection. Broad-spectrum antibiotic therapy is recommended because penetrating head injuries carry a high risk of intracranial infection and brain abscess formation [9].

Watertight dural closure is another critical step in surgical management to prevent CSF leakage and reduce the risk of meningitis. Autologous graft materials such as fascia lata or pericranium are commonly used for dural reconstruction [17].

Complications following penetrating brain injuries include intracranial hemorrhage, CSF leak, meningitis, brain abscess, traumatic pseudoaneurysm, and post-traumatic epilepsy [18]. In this case, early surgical intervention and meticulous infection control measures contributed to short-term clinical stability, although neurological recovery remained limited.

Our case highlights several important principles in the management of penetrating cranial trauma: careful preoperative imaging, controlled extraction of the foreign body along its trajectory, meticulous debridement, and effective dural repair. The patient demonstrated a stable neurological status following surgery, although residual hemiparesis persisted. This case also highlights the importance of avoiding premature removal of penetrating objects outside the operating room, as such objects may tamponade injured vessels and prevent catastrophic hemorrhage. This case was further complicated by the presence of a heavy retained object and delayed presentation, both of which increased the technical complexity of surgical management. However, this report is limited by the short follow-up duration and absence of vascular imaging, which may underestimate delayed vascular complications such as pseudoaneurysm formation.

## Conclusions

Low-velocity penetrating cranial trauma with retained foreign bodies is a rare but potentially life-threatening condition. Early recognition and prompt surgical intervention are crucial for preventing secondary brain injury and infection. Controlled removal of the foreign body under direct visualization, adequate debridement, and watertight dural repair remain the key principles of management. This case demonstrates successful acute surgical management and short-term clinical stability; however, long-term neurological outcomes remain uncertain and should be interpreted with caution.

## References

[1] (2019). Global, regional, and national burden of traumatic brain injury and spinal cord injury, 1990-2016: a systematic analysis for the Global Burden of Disease Study 2016. Lancet Neurol.

[2] Vakil MT, Singh AK (2017). A review of penetrating brain trauma: epidemiology, pathophysiology, imaging assessment, complications, and treatment. Emerg Radiol.

[3] Kataria R, Singh D, Chopra S, Sinha VD (2011). Low velocity penetrating head injury with impacted foreign bodies in situ. Asian J Neurosurg.

[4] Young L, Rule GT, Bocchieri RT, Walilko TJ, Burns JM, Ling G (2015). When physics meets biology: low and high-velocity penetration, blunt impact, and blast injuries to the brain. Front Neurol.

[5] Harrington BM, Gretschel A, Lombard C, Lonser RR, Vlok AJ (2021). Complications, outcomes, and management strategies of non-missile penetrating head injuries. J Neurosurg.

[6] Temple N, Donald C, Skora A, Reed W (2015). Neuroimaging in adult penetrating brain injury: a guide for radiographers. J Med Radiat Sci.

[7] Bodanapally UK, Saksobhavivat N, Shanmuganathan K, Aarabi B, Roy AK (2015). Arterial injuries after penetrating brain injury in civilians: risk factors on admission head computed tomography. J Neurosurg.

[8] Zyck S, Toshkezi G, Krishnamurthy S (2016). Treatment of penetrating nonmissile traumatic brain injury. case series and review of the literature. World Neurosurg.

[9] Kazim SF, Shamim MS, Tahir MZ, Enam SA, Waheed S (2011). Management of penetrating brain injury. J Emerg Trauma Shock.

[10] Medical Research Council (1976). Aids to the Examination of the Peripheral Nervous System. https://www.ukri.org/wp-content/uploads/2021/12/MRC-011221-AidsToTheExaminationOfThePeripheralNervousSystem.pdf.

[11] Skarupa DJ, Khan M, Hsu A (2019). Trends in civilian penetrating brain injury: a review of 26,871 patients. Am J Surg.

[12] Wu Y, Chen TG, Chen SM (2021). Trans-base and trans-vault low-velocity penetrating brain injury: a retrospective comparative study of characteristics, treatment, and outcomes. Chin J Traumatol.

[13] Castillo-Calcáneo JD, Bravo-Angel U, Mendez-Olan R, Rodriguez-Valencia F, Valdés-García J, García-González U, Broc-Haro GG (2016). Traumatic brain injury with a machete penetrating the dura and brain: case report from southeast Mexico. Int J Surg Case Rep.

[14] Santiago LA, Oh BC, Dash PK, Holcomb JB, Wade CE (2012). A clinical comparison of penetrating and blunt traumatic brain injuries. Brain Inj.

[15] Hofbauer M, Kdolsky R, Figl M, Grünauer J, Aldrian S, Ostermann RC, Vècsei V (2010). Predictive factors influencing the outcome after gunshot injuries to the head-a retrospective cohort study. J Trauma.

[16] Chowdhury FH, Haque MR, Hossain Z, Chowdhury NK, Alam SM, Sarker MH (2016). Nonmissile penetrating injury to the head: experience with 17 cases. World Neurosurg.

[17] Thammavaram KV, Benzel EC, Kesterson L (1990). Fascia lata graft as a dural substitute in neurosurgery. South Med J.

[18] Loggini A, Vasenina VI, Mansour A (2020). Management of civilians with penetrating brain injury: a systematic review. J Crit Care.

